# Enlightened prognosis: Hepatitis prediction with an explainable machine learning approach

**DOI:** 10.1371/journal.pone.0319078

**Published:** 2025-04-02

**Authors:** Niloy Das, Md Bipul Hossain, Apurba Adhikary, Avi Deb Raha, Yu Qiao, Md Mehedi Hassan, Anupam Kumar Bairagi

**Affiliations:** 1 Department of Information and Communication Engineering, Noakhali Science and Technology University, Noakhali, Chittagong, Bangladesh; 2 Computer Science and Engineering Discipline, Khulna University, Khulna, Khulna, Bangladesh; 3 Department of Computer Science and Engineering, Kyung Hee University, Yongin-si, Gyeonggi, Republic of Korea; Sunway University, MALAYSIA

## Abstract

Hepatitis is a widespread inflammatory condition of the liver, presenting a formidable global health challenge. Accurate and timely detection of hepatitis is crucial for effective patient management, yet existing methods exhibit limitations that underscore the need for innovative approaches. Early-stage detection of hepatitis is now possible with the recent adoption of machine learning and deep learning approaches. With this in mind, the study investigates the use of traditional machine learning models, specifically classifiers such as logistic regression, support vector machines (SVM), decision trees, random forest, multilayer perceptron (MLP), and other models, to predict hepatitis infections. After extensive data preprocessing including outlier detection, dataset balancing, and feature engineering, we evaluated the performance of these models. We explored three modeling approaches: machine learning with default hyperparameters, hyperparameter-tuned models using GridSearchCV, and ensemble modeling techniques. The SVM model demonstrated outstanding performance, achieving 99.25% accuracy and a perfect AUC score of 1.00 with consistency in other metrics with 99.27% precision, and 99.24% for both recall and F1-measure. The MLP and Random Forest proved to be in pace with the superior performance of SVM exhibiting an accuracy of 99.00%. To ensure robustness, we employed a 5-fold cross-validation technique. For deeper insight into model interpretability and validation, we employed an explainability analysis of our best-performed models to identify the most effective feature for hepatitis detection. Our proposed model, particularly SVM, exhibits better prediction performance regarding different performance metrics compared to existing literature.

## 1 Introduction

Nowadays, viral illnesses are becoming a serious hazard to human life. The liver plays a crucial role in the human body, serving as one of its major organs that carries out various essential and critical functions that are vital for our well-being. For instance, it is responsible for detoxification, metabolism, and the synthesis of essential proteins [1]. Several viral diseases can affect the liver, ranging in severity from mild, and acute to chronic conditions that can lead to serious liver damage. Moreover, liver disease (i.e., cirrhosis, viral hepatitis, and liver cancer) accounts for over two million deaths annually and accounts for 1 person out of every 25 deaths, indicating 4% of all deaths worldwide [2]. Hepatitis is considered to be one of the most common viral diseases responsible for liver damage. Hepatitis is also termed the inflammation of the liver caused by various infectious viruses, alcohol consumption, and several health problems due to noninfectious agents. Infections from viruses are considered to be the main reason behind hepatitis. There are 5 primary hepatitis viruses such as hepatitis A,B,C,D, and E virus [3]. Hepatitis B and **C** are the key reasons for liver cancer. Furthermore, chronic hepatitis C is a disease that persists over time and does not have an effective vaccine [4]. This condition frequently sets off serious infections in the body, including cancer, fibrosis, and liver cirrhosis [5]. The World Health Organization (WHO) claimed that over 354 million individuals in the globe are living with this [6]. In medical science, diagnosis of viral infections or illnesses becomes quite difficult because of the similarities with bacterial infections. Predictions might be a potential alternative solution as it provides an efficient way to identify patients at risk of serious complications. For example, patients with hepatitis B Virus (HBV) infection may be monitored more seriously compared to patients with hepatitis A virus (HAV) infections. This leveraging the advanced technologies of machine learning, we can develop efficient models that provide accurate predictions.

In the context of machine learning, various studies have been carried out regarding the prediction of hepatitis by analyzing patient records on various clinical tests. Different techniques of machine learning were investigated to determine their effectiveness in the classification of hepatitis. Lilhore et al.,2023 developed a Hybrid Predictive Model (HPM) that combined random forest (IRF) and support vector machine (SVM) techniques and achieved an accuracy of 96.82% with a precision of 98.93% [5]. The study employs a methodology that involves the selection of key features using Ranker and SMOTE techniques to improve the performance of HPM and addresses the shortcomings of random forest methodology. Although the research builds a strong model utilizing the same UCI Hepatitis C Virus (HCV) dataset, it still lacks competitive accuracy. Further, Safdari et al.,2022 applied data mining techniques and opted for Random Forest (RF), obtaining a comparatively better accuracy of 97.29% and a sensitivity of 90.99% [7] for hepatitis detection. Singh et al., 2022 employed supervised and unsupervised learning models such as K-means, Hierarchical clustering, DBMSCN, etc for their study on predicting hepatitis [8]. Additionally, they used a two-part dataset approach to obtain their results. In their case, Logistic Regression (LR) turned out to be a suitable model with an accuracy of 94.3%, albeit with lower precision and sensitivity. The research conducted by Hira et al. [9] indicates that when handling imbalanced datasets using an SVM model with an accuracy of 99.1% is a more effective and accurate choice for predictive modeling on imbalanced datasets compared to Multilayer Perceptron (MLP) with self-defined neural networks. Their study stands out for the impressive performance of the machine learning model despite having subpar quality in positive prediction. Ma et al., 2020 demonstrated a twisted performance of the XGBoost classifier with comparatively lower accuracy and precision. The proposed model of this study exhibited 98% sensitivity which means the model can accurately identify positive instances [10]. In a similar vein, Ahammed et al.,2020 [11] and Syafaâ et al., 2021 [12], turned to K-Nearest Neighbors (KNN) and Neural Networks (NN), respectively, showcasing accuracies of 94.40% and 95.12%. Further in [12], the author adopted the KNN model and incorporated different filter-based feature selection techniques including Gain Ratio Attribute Evaluation, Chi-Square Attribute Evaluation, ReliefF (RFAE), and Info Gain Attribute Evaluation (IGAE). In [13], Li et al.,2022 introduced a new method for detecting the likelihood of HCV infection called Cascade RF-LR (with SMOTE) using the ABC algorithm. The approach managed to detect the multiclass probabilities with an accuracy rate of 94.5%.

After reviewing the existing literature and considering the methodologies used in previous studies, our research aimed to build upon the purpose of accurate prediction by incorporating effective approaches. The proposed approaches involved a rigorous evaluation of different classification models including LR, RF, DT, SVM, and MLP. Our study brings up a sequential approach, especially in data preprocessing to refine the dataset for a more accurate and feasible prediction of hepatitis infection. This approach combines outlier detection, balancing data via synthetic minority oversampling technique (SMOTE), and others to enhance the integrity of the dataset, thus minimizing noise and biases (outlier removal technique) that may hinder model performance. Additionally, We implemented hyper-parameter tuning and cross-validation to optimize the performance of our models. Furthermore, we conducted a comparative analysis of different models and performed an explainability analysis to understand the models in handling the datasets. In contrast to other studies, we rely on a **comprehensive evaluation framework** that extends beyond accuracy, incorporating metrics such as precision, recall, and F1-score, essential for clinical decision-making.

Together with other aspects and approaches of our study, this multi-metric approach ensures the robustness and reliability of the machine learning models to be fitted in real-world clinical settings, particularly for the early classification of hepatitis infection. In brief, our study concentrated on enhancing the interpretability of our models as well as ensuring better performance for accurate prediction analysis.

Our key contributions are as follows:

- We proposed an effective classification machine learning model capable of predicting hepatitis and identifying stages of hepatitis C to aid healthcare professionals in making early and accurate diagnoses, potentially preventing permanent liver failures.
- We utilized the UCI dataset by performing significant data prepossessing techniques like SMOTE to mitigate bias and build more feature-focused models.
- We trained various classification models on unseen data by splitting data sets into train, test, and validation sets to evaluate the model’s generalization ability.
- Finally we analyzed the top-performing model for effectively pinpointing the most important features to identify hepatitis cases.

**Fig 1:**
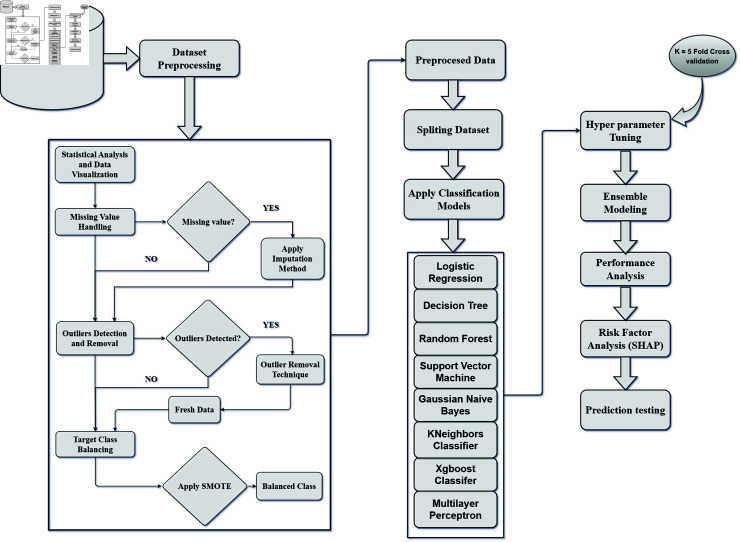
Proposed workflow of this study.

In the Materials and Methods section of this paper, we discuss the dataset preprocessing and describe the model used in this study. The Results section presents the performance of our model, followed by a comprehensive discussion. Finally, the Conclusion section summarizes the key findings and implications of our research. The overall procedure of this study is shown in Fig 1.

## 2 Materials and methods

### 2.1 Description of the dataset

In this study, we collected the dataset from the UCI Machine Learning Repository [14]. The collection contains laboratory and demographic data on patients with hepatitis C and blood donors. The data set can be characterized as multivariate and it consists of 615 instances and 12 features. Except for two features (Category and Sex) which are categorical, others are numerical. Table 1 is represented providing the basic description of the dataset including feature type, name, etc.

**Table 1:** Description of features in the dataset.

Feature	Representation	Data type	Description
Category	Category	Categorical	The target feature:
			’0 = Blood Donor’,
			’0s = Suspect Blood Donor’,
			’1 = Hepatitis’,
			’2 = Fibrosis’,
			’3 = Cirrhosis’
Age	Age	Numerical	Age of the patients
Gender	Sex	Categorical	Gender of the patients
Albumin	ALB	Numerical	Quantitative albumin
Alkaline phosphatase	ALP	Numerical	Quantitative alkaline phosphatase
Alanine transaminase	ALT	Numerical	Quantitative alanine transaminase
Aspartate aminotransferase	AST	Numerical	Quantitative aspartate aminotransferase
Bilirubin	BIL	Numerical	Quantitative bilirubin
Cholinesterase	CHE	Numerical	Quantitative cholinesterase
Cholesterol	CHOL	Numerical	Quantitative cholesterol
Creatinine	CREA	Numerical	Quantitative creatine
Gamma-glutamyl transferase	GGT	Numerical	Quantitative gamma-glutamyl transferase
Protein	PROT	Numerical	Quantitative protein

**Fig 2:**
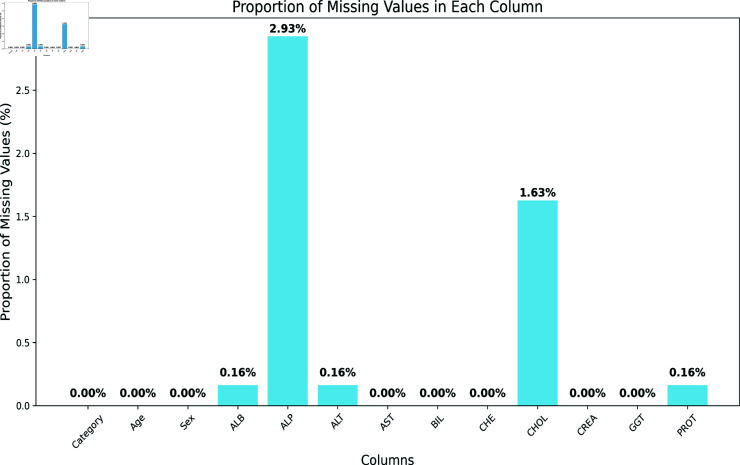
Missing values in data set.

### 2.2 Dataset pre-processing

Preprocessing is the process of converting the raw data into an organized format that can be utilized for machine learning (ML) model training. It is necessary to achieve more accurate model performance. The following data-prepossessing steps have been employed in this study:

#### Missing value handling

Handling missing values is a crucial aspect of data set pre-processing. Missing values can lead to biased results in a prediction analysis if not handled properly. The original data set contained a small percentage of missing values, as can be seen in Fig 2. The absent data points exhibit the Missing Completely at Random (MCAR) pattern. The imputation method is an efficient mechanism for handling missing values in this data type. To handle missing values, the mean imputation method has been employed. In this process, missing values were imputed using the mean value of other corresponding attributes. This particular method proved to be quite simple and effective for the data set as it provided minimal impingement.

**Fig 3:**
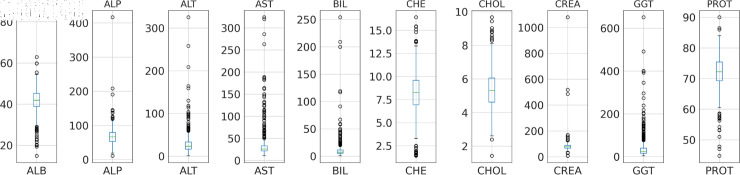
Box-plot distribution of data set for outlier detection.

#### Outlier detection and removal

Outliers can be defined as abnormal observations that are substantially different from other data points in a given dataset. Outliers significantly influence the performance of various machine-learning algorithms, which may lead to poor evaluation. Handling outliers includes the Detection and removal of the outlier values. The box plot in Fig 3 visualizes the distribution of data and identifies the data point falling outside of an expected distribution. Further, the Winsorization technique was used for the outlier removal from the dataset. This conservative approach alternates the value of an extreme observation with the nearest non-outlier values using the function *X_w_* so that extreme values have less effect on the estimated total [15]. A value modification strategy like winsorization can address many of the practical problems that arise in outlier robust inference [15]. This function calculates the value of *X_w_* based on the given inputs *X*, *T_l_*, and *T_u_*.


$$X_{w}=\min(\max(X,T_{l}),T_{u})$$

#### Target class balancing

**Fig 4:**
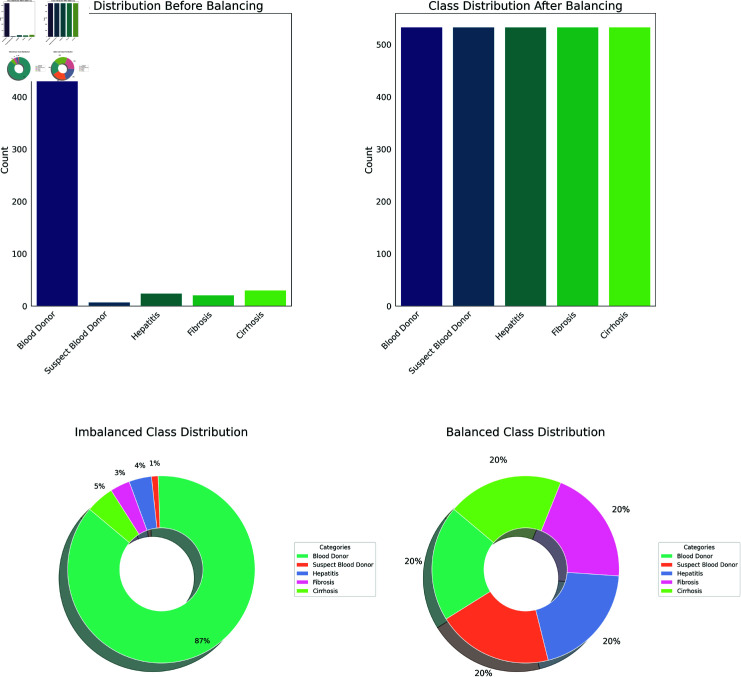
Class balancing using SMOTE.

The Synthetic Minority Oversampling Technique also known as SMOTE is considered to be an effective method for addressing class imbalances in machine learning [16]. To generate synthetic data it selects individual instances from the minority class, finds their closest neighbors, and produces fresh samples by interpolating between the chosen instance and its neighbors [17]. This process effectively increased the number of instances in the minority class, making a more balanced dataset and less prone to model bias. Fig 4 represents the quantitative percentage of values present in each class in the dataset. Further, it represents equal amounts of data after the class balancing is applied to the datasets.

### 2.3 Evaluation metrics for model performance

#### Confusion matrix of model evaluation

It is an *N* × *N* matrix or table used to assess the classification model’s effectiveness. The confusion matrix facilitates a comparative analysis between predicted and absolute values, providing a framework to weigh performance evaluation metrics (accuracy, precision, recall). For a binary classifier, four different parts of the confusion matrix are True Positive (TP), False Positive (FP), True Negative (TN), and False Negative (FN).

#### Accuracy

Accuracy can be referred to as the ability of a model to predict test data correctly. This performance metric is best suited for balanced target class variables. To calculate accuracy, the following formula can be used:


$$\text{Model’s Accuracy}=\frac{\text{Correct Predictions (TP+TN)}}{\text{Total Instances (TP+FP+TN+FN)}}$$

#### Precision

It measures the accuracy of positive predictions among the instances predicted as positive [18]. This can be defined as follow:


$$\text{Model’s Precision}=\frac{\text{Correctly Predicted Positive Instances (TP)}}{\text{All positive Instances (TP+FP)}}$$

#### Recall

Recall or Sensitivity measures the ability of a model to correctly detect positive instances from the entire set of original positive instances. The following formula is used for determining the recall of classification models:


$$\text{Model’s Recall}=\frac{\text{Correctly Predicted Positive Instances (TP)}}{\text{Total Actual Positive Instances (TP+FN)}}$$

#### F1-score

It provides a balance between precision and recall [19]. This minimizes both false positives and false negatives [20]. Thus it can be expressed by using the following formula:


$$F1\text{-score }=\frac{2(\text{Precision}*\text{Recall})}{\text{Precision}+\text{Recall}}$$

#### Specificity

Specificity identifies the model ability to accurately distinguish the actual negative cases from the pool of all negative cases. Below is the formula for specificity:


$$\text{Model’s Specificity}=\frac{\text{TN}}{\text{TN+FP}}$$

#### Cross validation

The method of k-fold cross-validation can be used to resample data for the assessment of the generalization ability of predictive models as well as to prevent over-fitting of data [21]. The technique has been applied in the data set of this study for the better utilization of classifiers, as explained in Fig 1. The dataset was split into k=5 folds for creating four folds as a training set and 1 for validation from the overall data set. Therefore, the average of performance metrics from each fold was calculated to obtain a single estimated performance of machine learning models on the validation set.

### 2.4 Machine learning algorithms.

In this research, we applied several supervised classification algorithms to our dataset. Each of these ML algorithms has its characteristics and ways of analyzing predictions and solving complex problems. This segment provides an introduction to these diverse algorithms along with their underlying principles.

#### Logistic regression

A supervised machine learning approach called logistic regression is a statistical method used to comprehend a binary or proportional response (dependent variable) based on one or more predictors [22]. Logistic Regression is commonly used in health science investigations as it is especially appropriate for models involving disease states (diseased or healthy) and binary decision-making (yes or no) [23]. The main role of Logistic regression is to maximize the likelihood of the observed data through an iterative process known as gradient descent. This particular algorithm is based on a sigmoid function known as the logistic function that converts any real value supplied into a probability between zero and one. This allows logistic regression to predict the probability of a binary outcome, such as the likelihood of a customer buying a product. The following formula defines the logistic function:


$$\sigma (z)=\frac{1}{1+e^{-z}}$$

The logistic function is often limited to values between 0 and 1 by the hypothesis of regression itself [24]. The sigmoid function can be written in terms of the hypothesis of *x* (*h_θ_*(*x*)):


$$\begin{array}{llll} z & =\beta_{0}+\beta_{1}x\; & & \;\\ \beta_{0} & =\text{Intercept}\; & & \;\\ \beta_{1} & =\text{Coefficient of the independent variable}\; & & \;\\ & \; & \end{array}$$

#### Decision tree

A decision-support hierarchical model also known as the Decision Tree algorithm uses a decision tree framework for mapping item observations to inferences about the item’s target value [25]. A decision tree is a tree-like flowchart that comprises internal nodes representing tests on attributes, branches reflecting outcomes of the test, and class labels assigned to each leaf node (or terminal node) [25]. These decision nodes split up the data based on minimum entropy or maximum information gain. The entropy and information gain formulas are given as follows:


Entropy,E(S)=−∑i=1Cpi log ⁡ 2piInformation Gain,IG(S,A)=E(S)−∑v∈A|Sv||S|E(Sv)

#### Random forest

The Random Forest algorithm is considered to be a potent ensemble-based classifier that enhances model performance by taking the average of several decision trees on different subsets of the provided data set. It is made up of a set of decision trees produced by the bagging method without being pruned, creating a “forest” of classifiers that vote for different classes [26]. The number of trees (ntree) in the forest and the number of randomly chosen features/variables utilized for assessment at each tree node (mtry) are the two parameters that must be defined to train an RF [26]. RF utilizes the same metrics as Decision Tree (DT) classifiers.

#### Support vector machine

Another example of a supervised machine learning technique is the Support Vector Machine, which is employed in problems related to regression and classification. Finding the hyperplane that best divides the data into distinct classes while maximizing the margin between classes is the aim of support vector machines (SVMs). This study applied a non-linear SVM, specifically the Radial Basis Function (RBF) kernel for prediction analysis. The RBFkernel [27] is defined as:


$$\text{RBF Kernel},K(y,y^{\prime })=\exp\left(-\frac{∥y-y^{\prime }{∥}^{2}}{2\sigma^{2}}\right)$$

Here:

- ∥*y*−*y^′^*∥: The Euclidean distance between two data points.
- *σ*: The width parameter of the kernel.

#### Gaussian NB classifer

A collection of probabilistic classification methods known as “Naive Bayes classifiers” are based on Bayes’ Theorem with the assumption that features or characteristics are conditionally independent. Gaussian Naive Bayes states the assumption of features based on Gaussian distribution. This algorithm models the probability using a Gaussian Distribution’s probability distribution function (PDF) *P* ( *x* ∕ *C* )  for each feature *x* of a given class *C*. The PDF of a Gaussian Distribution is given by [28]:


P(x|μ,σ)=12πσ2 exp ⁡  (−(x−μ)22σ2)

#### Kneighbors classifier

The Kneighbors Classifier or KNN is well known for its simplicity and versatility among many supervised machine learning models. The classifier primarily relies on the distance or similarity that can be found between the tested examples and the trained examples [29]. Using the selected distance metric, a given data point in the training data set is matched with its k nearest neighbors in the case of this classifier. When it comes to classifications, the class that appears the most frequently is allocated to the data point, and the class label is decided by a majority vote among the data point’s k nearest neighbors. The distance metric equation can be defined as:


$$D(y,y^{\prime })=\sqrt{\overset{n}{\underset{i=1}{\sum }}{\left(y_{i}-y_{i}^{\prime }\right)}^{2}}$$

Here:

- *n*: the number of features.
- *y_i_* and $y_{i}^{\prime }$: the *i*-th features of *y* and *y^′^* respectively.

#### XGBoost classifer

Extreme Gradient Boosting (XGBoost) is a highly effective tree-based classification method that has been increasing in popularity for data classification recently [30]. This classifier produces several weak learners, usually decision trees, and then aggregates their predictions to get a final prediction using the ensemble learning approach. It also implies the idea of gradient boosting, in which every weak learner is trained to fix the mistakes made by the previous ones. Compared to other supervised classifier algorithms like RF, DT, and Gradient Boost, the XGBoost classifier performs better for both regression and classifications in terms of execution speed and model performance. For effective prediction on multiclass classification, the study employed the XGBoost classifier model.

#### Multilayer perceptron

The most popular kind of neural network is the multilayer perceptron [31]. Three different kinds of layers make up the perceptron algorithm: input layers, output layers, and hidden layers. Just like the human brain, every neuron in the hidden layer is connected to every other neuron in the next layer. The output layer is responsible for tasks like prediction and classification. The combination of the input and output layers is called a perceptron. These perceptrons are arranged in a feedforward manner, which involves propagating the input through the network by performing the weighted sum and activation function calculation for each neuron. The final output is provided by the nodes (neurons) in the output layer.

## 3 Result and discussions

### 3.1 Exploratory Data Analysis (EDA)

The term “Exploratory Data Analysis” or “EDA” refers to the methods for analyzing and investigating data sets to gain insights into the main features of the data. It helps to identify the patterns of data by employing data visualization methods. EDA is primarily used to explore a data set’s relationship between variables or features.

#### Descriptive statistic

One of the efficient methods used in EDA is the summary statistics of the data set which comprises variability metrics (e.g., standard deviation, variance, etc.) and measures of central tendency (e.g., mean, mode, and median). The descriptive statistics of our data set are presented in Table 2. This descriptive statistic provides a comprehensive overview of the data set used in this research. According to Table 2, there are 5 distinct categories of patients ranging from 0 to 4 and average age and standard deviation are respectively 47.41 and 10.0. Additionally, the statistical approach delves into the valuable properties of other numerical features such as their average, range of values, skewness, etc. For example, the average bilirubin level is 2.18769, and alanine aminotransferase (ALT) levels range from 2.23 to 4.1447. The table also demonstrates the number of missing values for each feature which required for data pre-processing. In brief, this descriptive statistic table highlights the need for a nuanced understanding of the data set.

**Table 2:** Descriptive statistic of the data set.

Attributes	Distinct	Mean	Min	Max	Std	Var	Skew	K-missing values	Zeros
Category	5	0.3869	0	4	1.0523	1.11E+00	2.6097	0	533
Age	49	47.4081	19	77	10.0551	1.01E+02	0.2671	0	0
Sex	2	0.3869	0	1	0.4875	2.38E-01	0.4651	0	377
ALB	146	41.6966	32	49	4.4812	2.01E+01	-0.3479	1	0
ALP	362	67.1907	37	104	18.1071	3.28E+02	0.2485	18	0
ALT	287	3.1858	2.23	4.14	0.4895	2.40E-01	0.0682	1	0
AST	248	0.2062	0.21	0.21	0.00006	3.97E-09	0.0862	0	0
BIL	150	2.1877	1.39	3.22	0.5007	2.51E-01	0.4097	0	0
CHE	350	8.1939	4.52	11.39	1.843	3.40E+00	-0.1611	0	0
CHOL	262	5.3556	3.62	7.29	0.9946	9.89E-01	0.1791	10	0
CREA	83	78.3082	55.2	106	14.0062	1.96E+02	0.3033	0	0
GGT	306	3.3235	2.42	4.73	0.6546	4.29E-01	0.6493	0	0
PROT	153	72.2663	64.1	80.3	4.3112	1.86E+01	-0.0407	1	0

#### Data distribution and visualization

**Fig 5:**
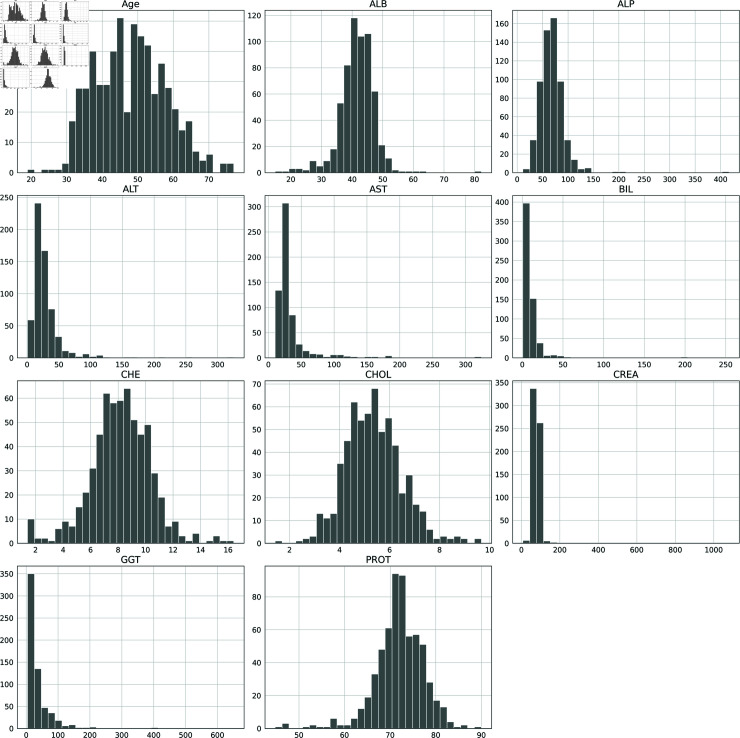
Frequency distribution of the data set.

Understanding the distribution of data in a data set is essential for prediction analysis as it explains the nature of data. Further, it depicts how the variability of different features occurs within the dataset. To comprehend and analyze the data distribution, we used data visualization in this section. Visualization of data includes the following plots and diagrams:

**Histogram analysis:** The histogram depicts the frequency distribution of numerical features of the data set. The vertical axis (Y-axis) represents the number of patients falling inside each characteristic, while the horizontal axis (X-axis) shows the range of values for each feature. As shown in Fig 5, most of the features have right-skewed distributions except for albumin (ALB), cholinesterase CHE, and proteins (PROT). In the case of negatively skewed distributions, most data points are concentrated on the right slide indicating the lack of extreme values on the left side.

**Fig 6:**
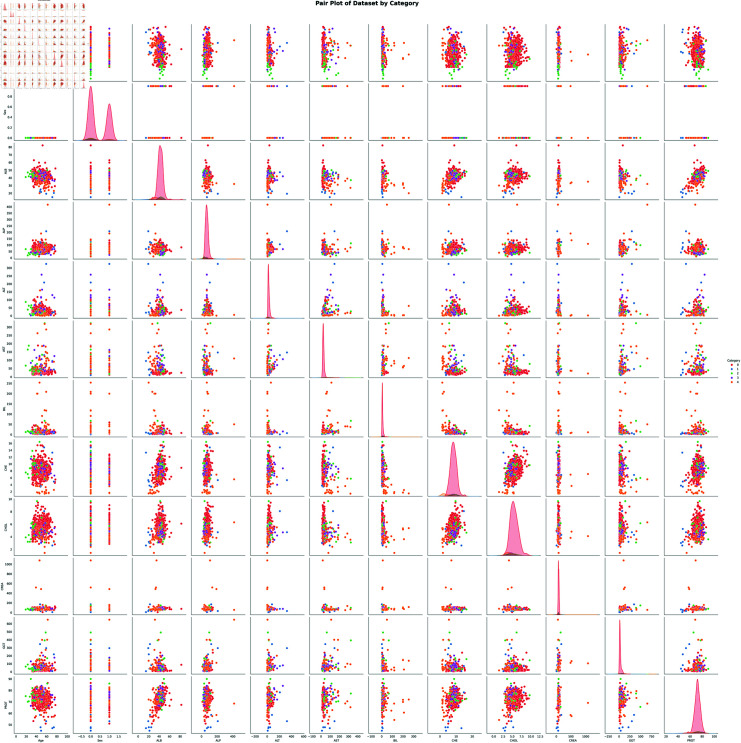
Pair wise relationship of multiple variables in data set.

**Fig 7:**
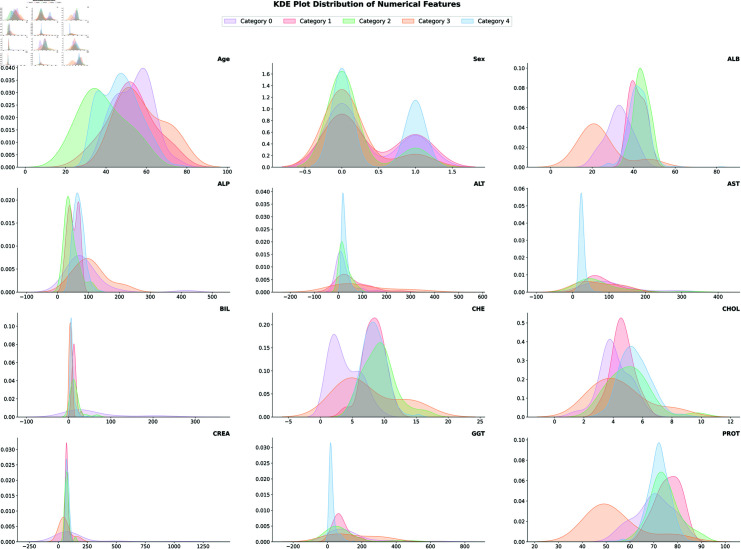
KDE plot distribution of data.

**Pair plot analysis:** A pair plot visualizes the relationship between pairs of variables which can be utilized to spot the patterns and potential correlations in a set of data. As shown in Fig 6, the pair plot has a grid of scatter plots and diagonal elements showing the distribution of features in the form of KDE plots as shown in Fig 7. In Fig 6, the five distinct colors symbolize the five categorical values, while the X and Y axes show the numerical aspects of the data set. This color-coded approach helps in distinguishing between categories and identifying patterns within data. The pair plot reveals several intriguing relationships among the liver function tests including correlation, and skewness. For example, a strong positive correlation between creatinine (CREA) and cholesterol (CHOL) can be used to identify patients with alcoholic liver disease. Outliers among the features become apparent in the off-diagonal side of Fig 6. Additionally, the scarcity of linearity in these relationships suggests the complex nature of interactions within the dataset, warranting further investigation into potential contributing factors.

**KDE plot:** Using the positions of each sample point, the kernel approach generates a smooth approximation of the probability density function and more persuasively supports multimodality [32]. Kernel Density Estimation applies a kernel function on each data point in the data set producing a smooth estimation of the density by summing the functions. The kernel function representing the bell-shaped curve decays as it moves away from the data point. The KDE plot demonstrated in Fig 7 provides an insight into the distribution of key numeric variables across different classes of target variables. The legend in the plot mapped ’Category’ values into different colors. In brief, the plot shows that most of the data points fall into the categories of 0 (blood donor), 1 (suspect blood donor), 2 (hepatitis), 3 (fibrosis), and 4 (cirrhosis). As seen in Fig 7, the age variable reveals unimodal distribution across different categories of target variables. Distinct peaks in BIL, and CREA levels indicate significant differences in severity across the target variables.

**Fig 8:**
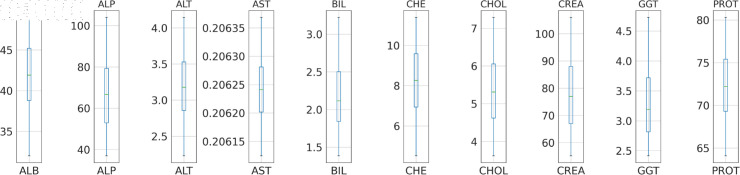
Boxplot: without outliers.

**Fig 9:**
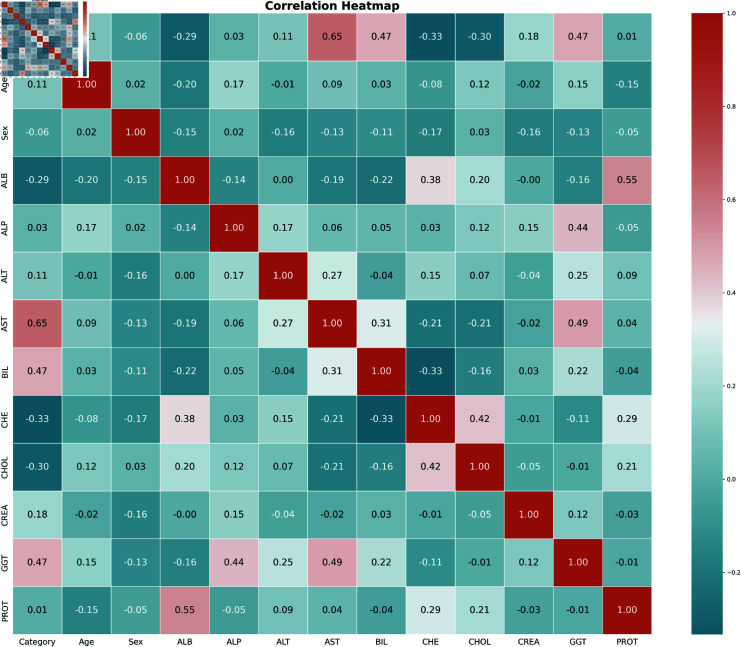
Heatmap of the data set.

**Fig 10:**
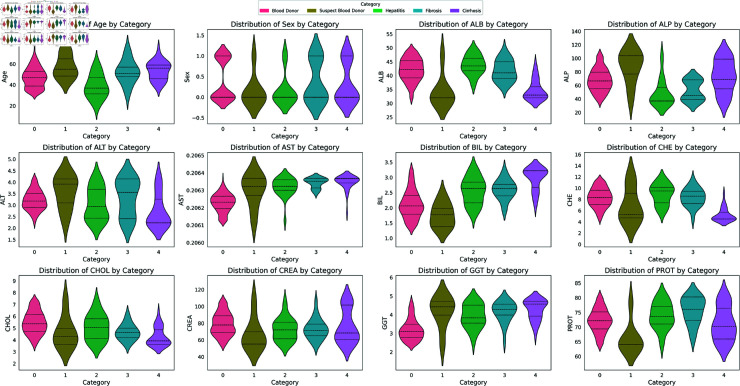
Target class distribution among features.

**Box plot:** The box plot uses the median, the approximate quartiles, and the lowest and highest data points to illustrate the three properties of data distribution: level, spread, and symmetry [33]. The box plot shown in Fig 8 illustrates the distribution of numerical features or liver function tests, with each test represented by a box with whiskers. The whiskers extend to cover the remaining 25% on either side of the box, which represents the middle 50% of the data (interquartile range). As seen in Fig 3, outliers are plotted as individual points, and the horizontal line within each box indicates the median of each test.

#### Correlation analysis

The study utilized a heatmap to emphasize the correlation between multiple dataset variables. The correlation heatmap visualized in Fig 9 is a color-coded matrix in which each row represents each variable and column and the cell shows the correlation between them. The degree and direction of the association are represented by different color hues where stronger relationships are shown by deeper color shades. The diagonal line portrays the relationship of variables with itself and it shows a positive correlation. The further interpretation of the heatmap leads to a clear image of a strong correlation between ALB and PROT features. As seen in Fig 9, other features also visualized strong correlations like CGT with AST, ALP, and CHE with ALB. The correlation heatmap enhances the machine learning model by providing decision-making observations on the relations among multiple numerical features.

#### Distribution of target variable

Fig 10 depicts the distribution of the target class ‘category’ across different features. The width of each curve is around the frequency of data points in each region. The figure illustrates that the majority of the attributes exhibit a distinct distribution over the target class’s various categories. For instance, feature ALT exhibits a wide and more spread distribution in categories 1, 2, and 3 compared to other categories. On the other hand, the wide distribution depicts a broad range of PROT test results observed among hepatitis C patients as well as Fibrosis patients.

### 3.2 Performance evaluation:

The study proceeded to preprocess the original data set and then evaluated various machine-learning models. The final step in preprocessing involved scaling and class balancing (as detailed in Sect 2.2.3). The preprocessed data set was then applied to several machine-learning models including classifiers.

We divided our data set into training, validation, and test sets using the train_test_split function. Initially, 70% of the data was utilized for training and 30% was used for testing and validation. A 50:50 split was used to equally divide the validation test set into distinct validation and test sets. For assessing the algorithm’s performance, several performance evaluation criteria were taken into account, including accuracy, precision, recall, AUC score, etc.

The section outlines the outcomes of the unique and relative approaches implemented for our study of the classification of hepatitis and liver diseases. This section provides a comparative analysis of the best results achieved by performing machine learning models on all features, incorporating hyper-parameter tuning, cross-validation, and employing ensemble modeling.

#### Model performance analysis using all features

**Fig 11:**
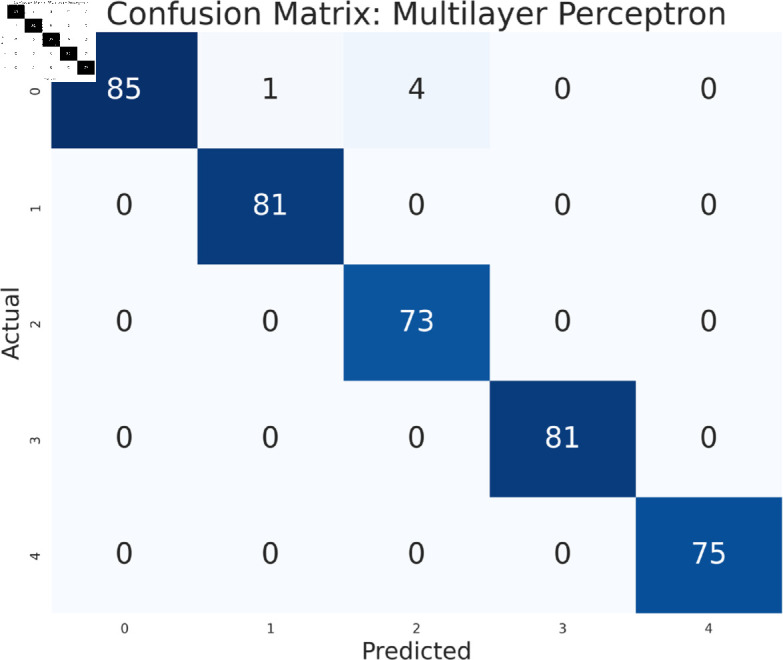
Confusion matrix of multilayer perceptron model.

**Fig 12:**
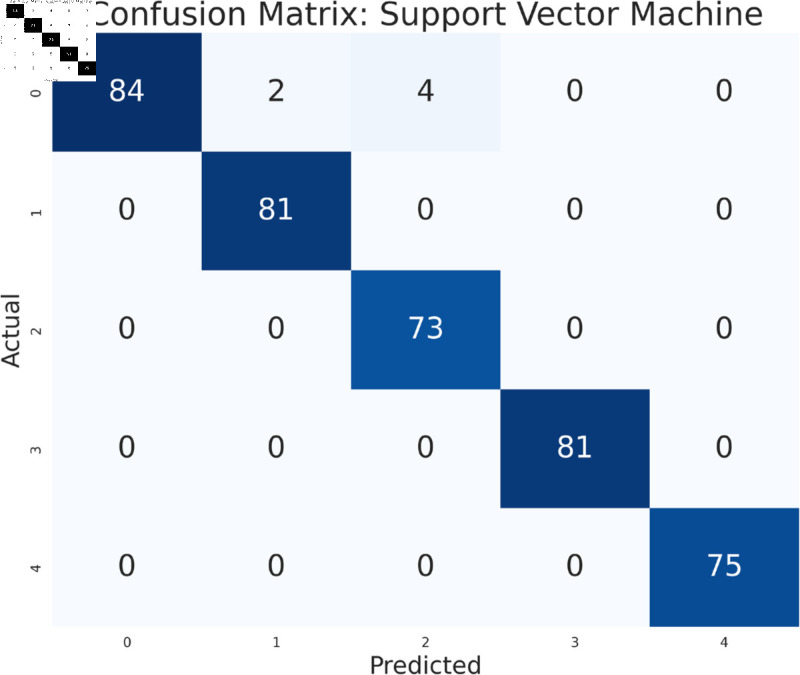
Confusion matrix of support vector machine model.

**Fig 13:**
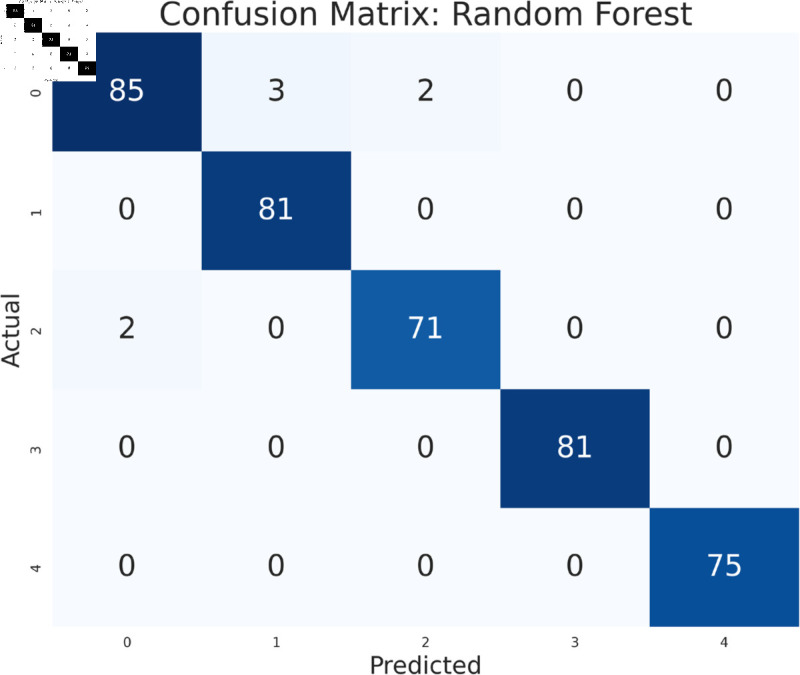
Confusion matrix of random forest model.

**Fig 14:**
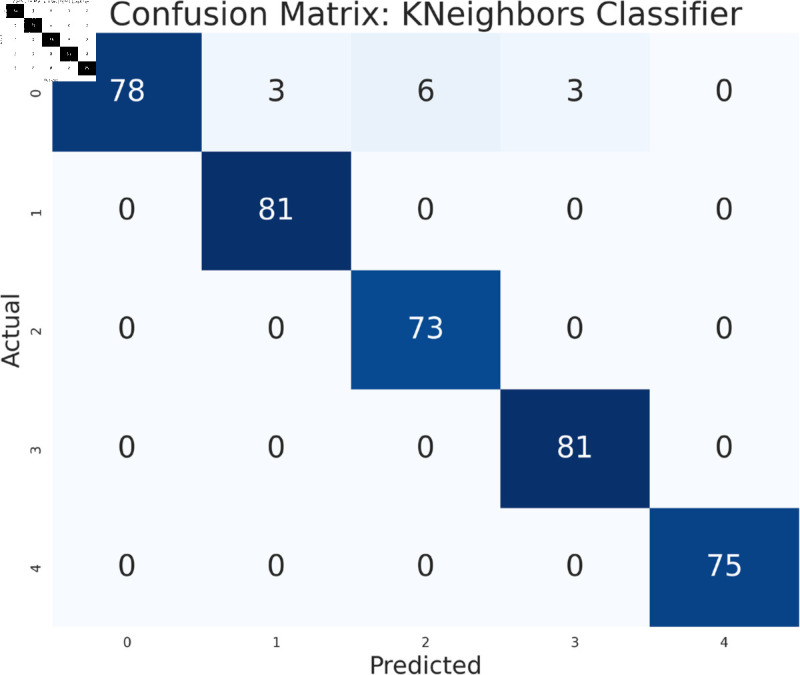
Confusion matrix of KNeighbors classifier.

**Fig 15:**
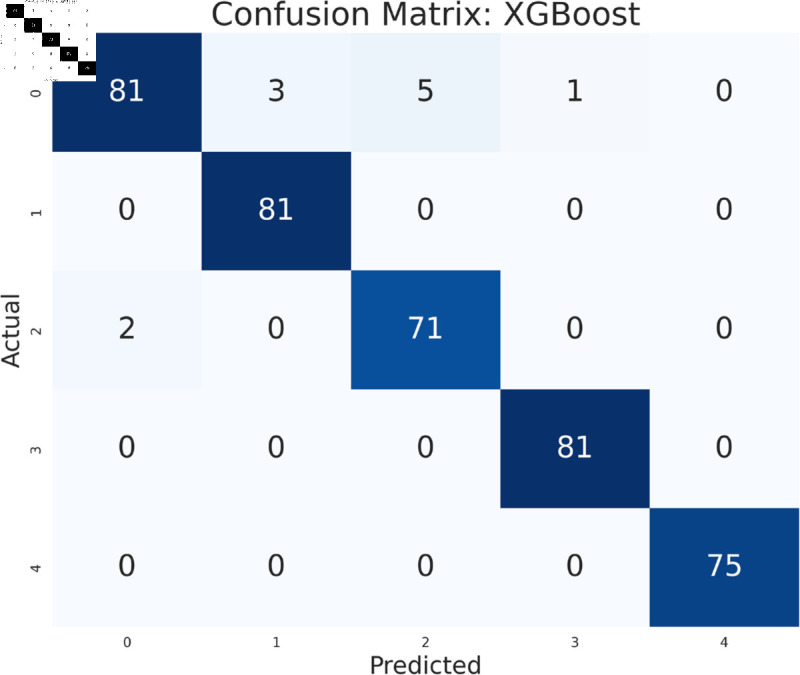
Confusion matrix of XGBoost model.

**Fig 16:**
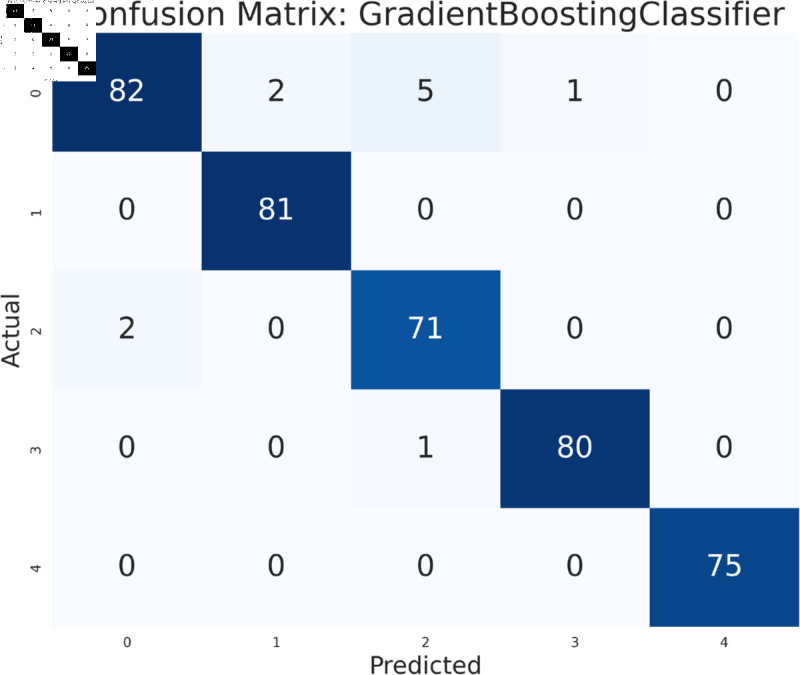
Confusion matrix of gradient boosting classifier.

**Fig 17:**
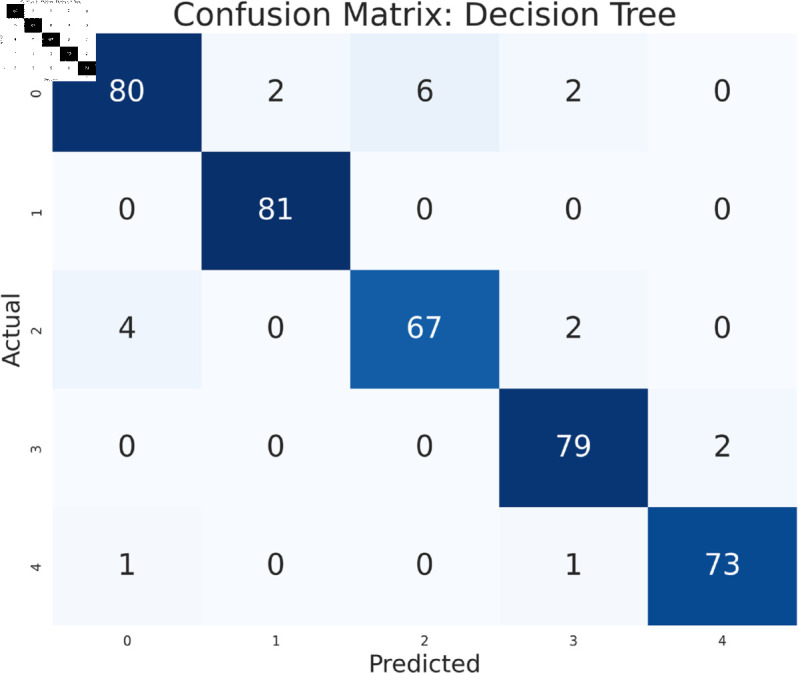
Confusion matrix of decision tree.

**Fig 18:**
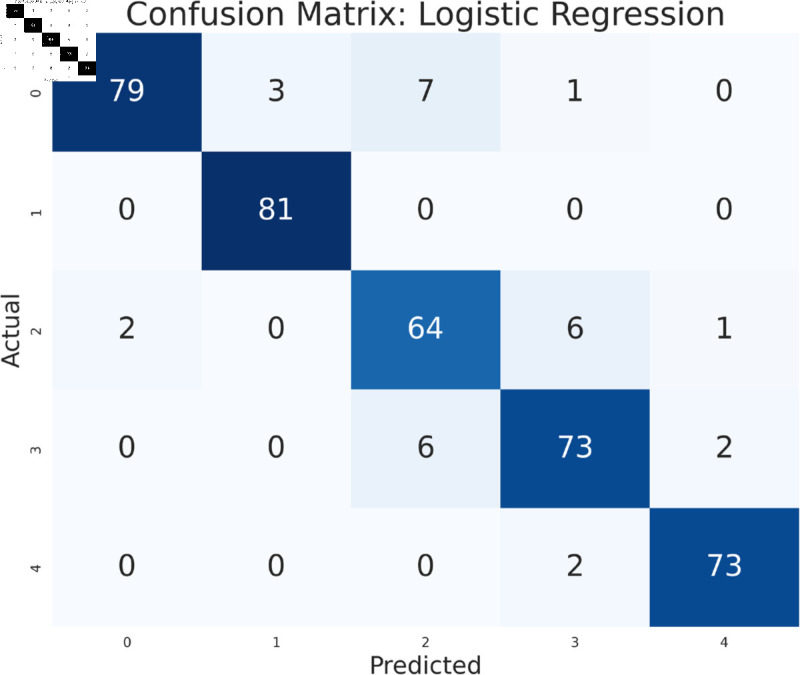
Confusion matrix of logistic regression.

**Fig 19:**
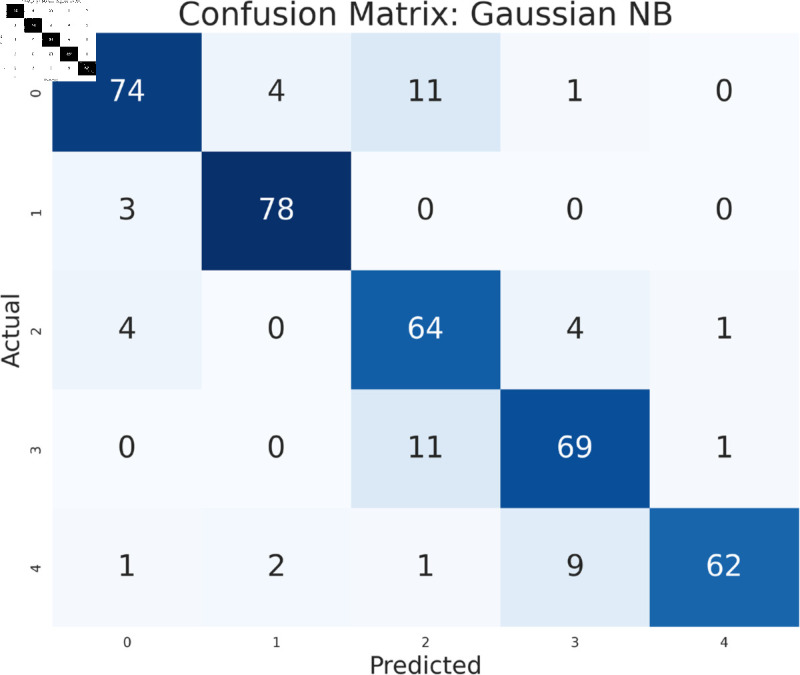
Confusion matrix of Gaussian NB.

**Fig 20:**
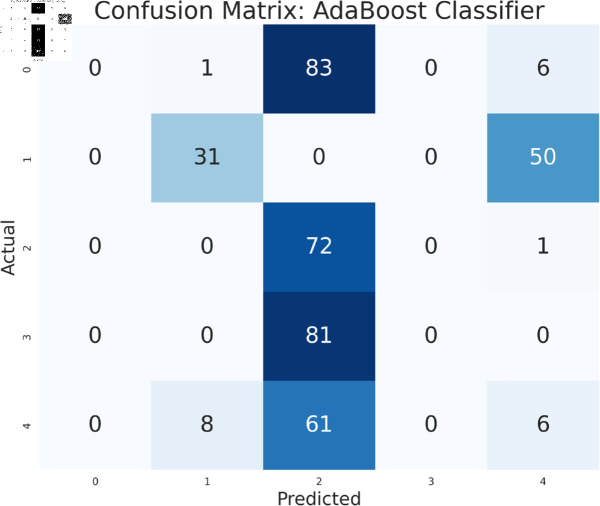
Confusion matrix of AdaBoost classifier.

The study has analyzed the dataset using a variety of machine learning models. This particular approach seeks to assess the performance of machine learning algorithms by using all available features in the data set. We simply applied the algorithms to our data set without performing any feature selection or dimensionality reduction technique before model training. Following that, the performance of different models is contrasted using performance evaluation criteria, such as f1-measure, accuracy, precision, and recall. As we can see in the Table 3, the models achieve a good score in accuracy which indicates that the models are effective in classifying the data. Among all the models, Random forest, SVM, XGBoost, and multilayer perceptron have been at the peak of accuracy. However, high accuracy does not always guarantee efficient performance, especially in the case of prediction. Therefore, Random Forest, Support Vector Machine, and Multilayer Perceptron consistently demonstrate strong performance across all models considering other evaluation metrics like precision, recall, and F1-measure. This implies a good balance between identifying true positives and avoiding false positives and false negatives, making them promising candidates for classification. The Multilayer Perceptron achieved the highest score of 99% almost in every metric except a log-loss of 10.422. The Random Forest outperformed the Multilayer Perceptron and Support Vector Machine in vain, as evidenced by its log-loss of 20.96, depicting the uncertainty in the model’s prediction. As seen in Table 3, the AdaBoost Classifier, despite having a low overall accuracy, shows a higher precision of 22% than recall, suggesting that it makes a correct prediction but it misses a significant number of positive instances. Another example of lower performance was Gaussian NB in terms of accuracy and F1-measure, which might be due to its assumption of feature independence. On the basis of performance, we can conclude that Random forest (RF), Support Vector Machine (SVM), and Multilayer Perceptron (MLP) are the top performers for classification tasks while AdaBoost classifier and Gaussian NB exhibit poor performance. The confusion matrix of all algorithms are shown in Figs 11, 12, 13, 14, 15, 16, 17, 18, 19, and 20 to understand the performance more precisely. Based on these figures, a better understanding of model performance can be drawn as the matrix provides insight into the model’s performance relative to actual classification. The confusion matrix of AdaBoost classifier reveals significant misclassifications, especially between class 0 and class 1 where 83 instances of class 0 are predicted as class 1. Class 3 is frequently misclassified as class 0, depicting the poor performance of the model. In contrast, the matrix reflects the strong performance of MLP, with most classes being classified correctly. Not only that, the diagonal dominance in the confusion matrix also highlights the minimal misclassification of class 2.

**Table 3:** Experimental results of ML algorithms using all features.

Models	Accuracy(%)	Macro-avg precision(%)	Macro-avg recall(%)	Macro-avg F1-score(%)	AUC	Log-loss	MCC
Logistic regression	92.50	92.43	92.58	90.65	0.99	7.6	0.91
Decision tree	94.75	94.75	94.90	94.81	0.97	29.74	0.94
Random forest	98.25	98.28	98.34	98.29	1.00	20.96	0.98
Support vector machine	98.50	98.48	98.67	98.53	1.00	6.03	0.98
Gaussian NB	86.75	87.33	86.81	86.79	0.98	14.19	0.84
AdaBoost classifier	27.25	22.25	28.98	19.77	0.64	5.02	0.15
GradientBoosting classifier	97.25	97.24	97.43	97.92	1.00	7.49	0.97
XGBoost	97.25	97.24	97.45	97.30	1.00	7.41	0.97
Multilayer perceptron	98.75	98.72	98.89	98.77	1.00	10.42	0.99

#### Model performance optimization

The study employed hyperparameter tuning to enhance the predictive nature of the classifying models. Due to the fact that the performance of machine learning models is directly impacted by the choice of ideal hyperparameters [34]. Hyperparameter refers to parameters that cannot be updated during the training of machine learning [35]. These hyperparameters work as a predefined setting that controls the learning process of machine learning models. Proper tuning of these parameters plays an essential role in optimizing the model’s accuracy and generalization. Therefore, to optimize the overall performance of models, key hyperparameters were tuned for each model as presented in Table 4. For instance, the hyperparameters: the maximum depth of the tree (max_depth), the least number of samples needed to split a node (min_samples_split), and the number of trees (n_estimators) are optimized for Random Forest. Similarly, we adjusted the hyperparameters for Multilayer Perceptron: the activation function, L2 regularization term (alpha) to prevent overfitting, and sizes of hidden layers (hidden_layer_sizes) to define the neural network’s architecture. The best estimator for the MLP Classifier we obtained was: The tangent hyperbolic function for activation (tanh), alpha value of 0.001, and two hidden layers with 50 neurons each i.e. hidden_layer_sizes (50,50). Another noticeable change made during hyperparameters tuning was to the Support Vector Machine model where we explored the various combinations of hyperparameters to identify the optimal configuration for the model. SVM has some default parameters as follows [36]:

- *C*: 1.0 (the regularization factor to balance between misclassification and margin)
- Kernel: ‘linear’ or ‘rbf’ (type of kernel to be used in the algorithm)
- Gamma value: ‘scale’ (kernel coefficient)

The parameters we tuned with different ranges of values are:

- Values for *C*: [0.1, 1, 10]
- Available kernels: [‘linear’, ‘rbf’]
- Gamma value: ‘auto’

We applied the grid search approach using 5-fold cross-validation that determines the ideal set of hyperparameters by methodically searching through a given hyperparameter space. The cross-validation technique was discussed earlier in Sect. 2.3.7. In the case of SVM, we identified that regularization factor C with a value of 10 and The Radial Bass Function, or RBF, significantly affects the model’s overall performance.

**Table 4:** Optimized performance of models using hyper-parameter tuning.

Model	Hyperparameters tuned	Accuracy (%)	Std Dev (Acc)	Precision (%)	Recall (%)	F1-measure (%)
Logistic regression	Regularization strength (C): 1,					
	Penalty: ‘l2’	92.75	± nan	92.80	92.83	92.75
Decision tree	Maximum depth: None,					
	Minimum samples split: 2	96.75	± 0.011053	97.25	97.23	97.22
Random forest Maximum depth: None,	Number of estimators: 200,					
	Minimum samples split: 2	99.00	± 0.006695	99.00	98.99	98.99
Support vector machine	Regularization strength (C): 10,					
	Kernel: ‘rbf’	99.25	± 0.009774	99.27	99.24	99.24
Gaussian Naive Bayes	None	88.75	± 0.014427	88.99	88.85	88.77
K-Neighbors classifier	Number of neighbors (n_neighbors): 3	98.75	± 0.005134	98.78	98.73	98.73
AdaBoost classifier	Learning rate: 0.01,					
	Number of estimators: 50	72.50	± 0.035213	76.66	72.23	70.81
Multilayer perceptronActivation: ‘tanh’,	Hidden layer sizes: (50, 50),					
	Alpha: 0.01	99.00	± 0.004601	99.06	98.98	99.00

As seen in Table 4, the performance scores of each model have been improved demonstrating the effectiveness of hyperparameter tuning and cross-validation. A noticeable change can be observed in the performance of the AdaBoost Classifier. The hyperparameter tuning brought a significant increase in the accuracy from 28% to straight 72.5% but with a decrease in other metrics. The Gaussian NB also performed a little better than the previous approach. For instance, the model achieves an increase of nearly 2% in every evaluation metric which can be considered significant for precision and recall. Random Forest and SVM have been the top performers from the previous approach, yet these classification algorithms slightly improved every metric. These models perform nearly at the same pace with an accuracy of 99% and 99.25%.

Hyperparameter tuning combined with cross-validation effectively improved the performance of the AdaBoost classifier and Gaussian Naive Bayes although the technique only brought minimal improvement in other models. As per the study, Hyperparameter tuning can be used to obtain an optimal improvement through the evaluation of models. Based on our following analysis, it has been determined that the Support Vector Machine (SVM) is the most suitable candidate for classification tasks. Table 4 also shows that both the Random Forest (RF) and the Multilayer Perceptron (MLP) exhibit comparable performance.

#### Model performance enhancement through ensemble modeling

Prediction analysis requires sustainable performance from the machine learning models to provide accurate analysis. Therefore, we have used the ensemble modeling approach, specifically the voting classifier,by evaluating to enhance the performance by combining multiple models. The goal is to produce more accurate and robust predictions than individual models. A voting classifier is suitable for merging the same or conceptually different models for prediction using a voting scheme [37]. A voting classifier has two types of schemes: hard voting and soft voting. When using soft voting, the base models must have the ability to provide predicted probabilities. By combining the predictions of various models, the voting classifier tends to yield more favorable outcomes than other individual base models [37]. We utilized the three top-performing models - Random Forest, Multilayer Perceptron, and Support Vector Machine. Each model contributes its confidence level (probability) for each class. The ensemble approach then combines the probabilities to determine the final prediction.

As shown in Table 5, our use of the ensemble modeling technique has been proven to be successful in enhancing predictive performance with the highest score in each and every metric.

**Table 5:** Ensemble model performance comparison.

Models	Accuracy (%)	Precision (%)	Recall (%)	F1-score (%)
Random forest	99.00	99.00	98.99	98.99
Support vector machine	99.25	99.27	99.24	99.24
Multilayer perceptron	99.00	99.06	98.98	99.00
**Ensembled model**	**99.51**	**99.54**	**99.50**	**99.51**

### 3.3 Explainability analysis

The study highlights the interpretation of top-performing machine-learning models to gain insight into the decision-making process. We have utilized the model agnostic interpretability methods to explain the feature impacts on the model’s prediction.

**Fig 21:**
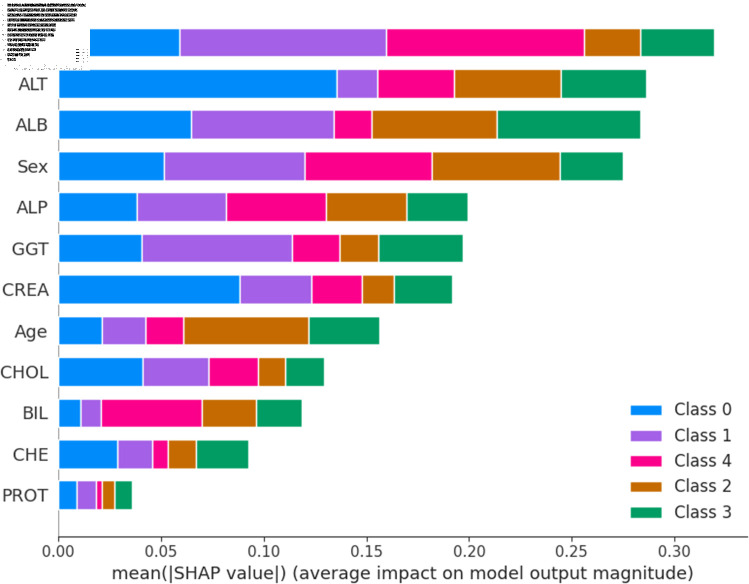
Features impacts of model output for support vector machine.

**Fig 22:**
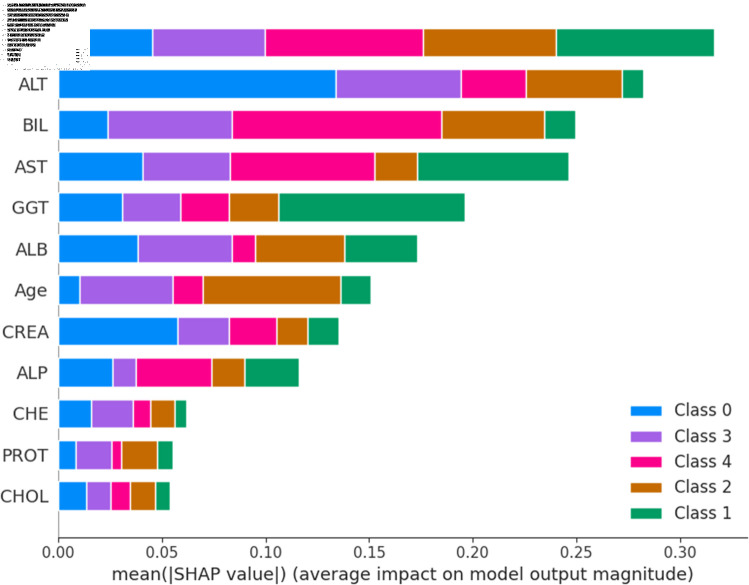
Features impacts of model output for random forest.

**Fig 23:**
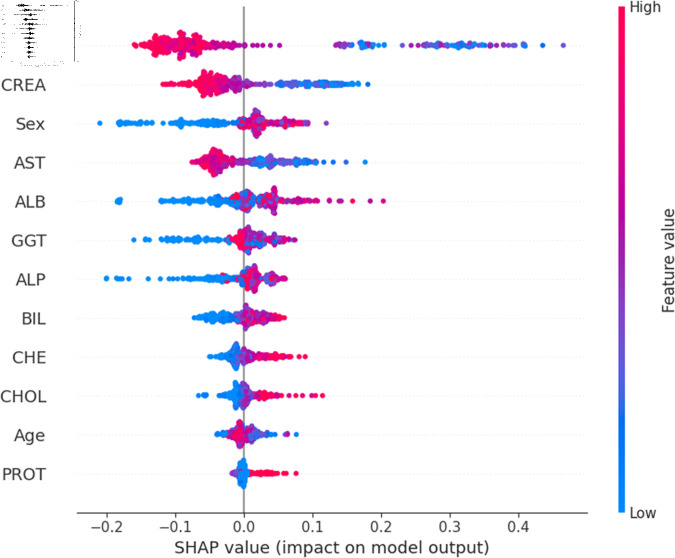
Explainability analysis of random forest model for blood donor.

**Fig 24:**
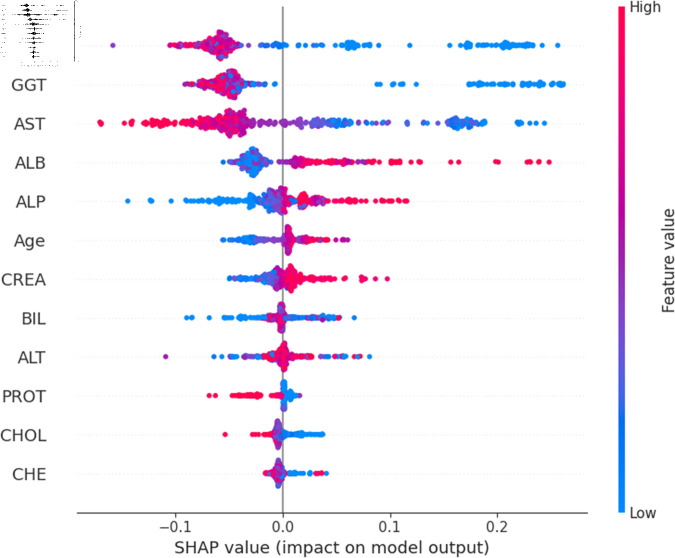
Explainability analysis of random forest model for suspect blood donor.

**Fig 25:**
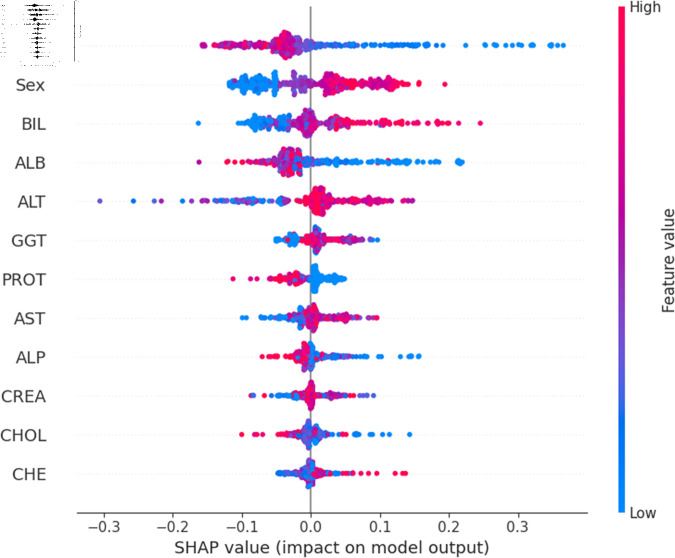
Explainability analysis of random forest model for hepatitis.

**Fig 26:**
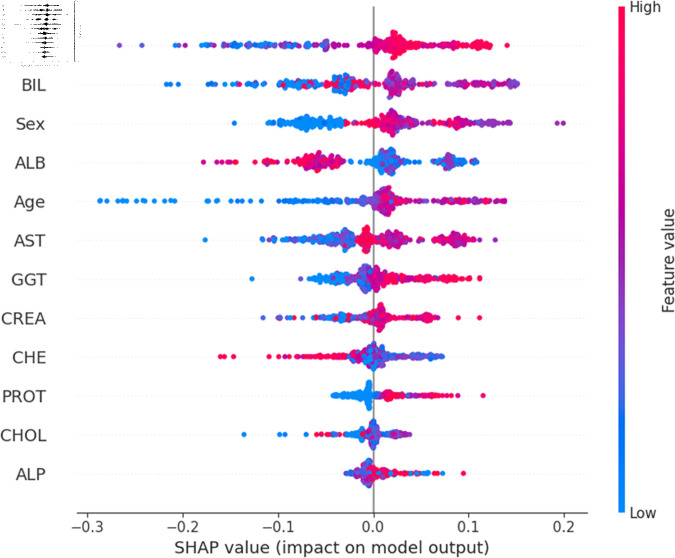
Explainability analysis of random forest model for fibrosis.

**Fig 27:**
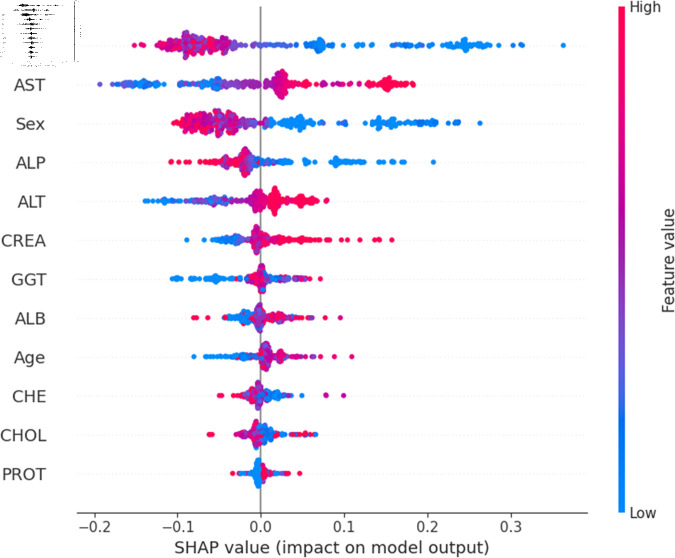
Explainability analysis of random forest model for cirrhosis.

**Fig 28:**
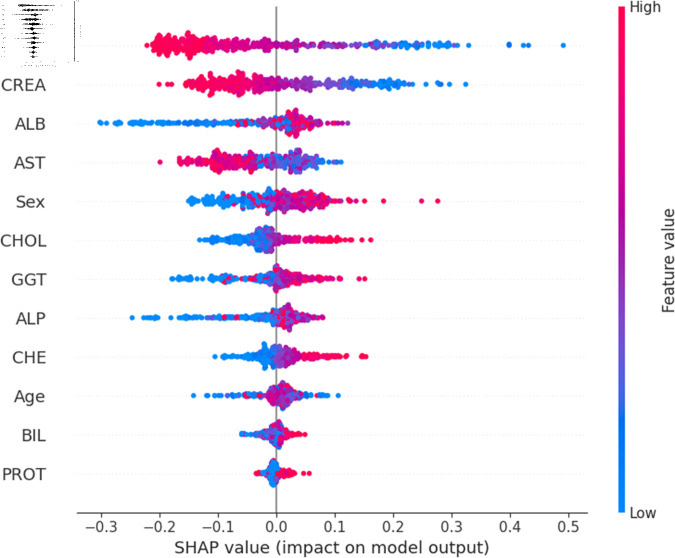
Explainability analysis of support vector machine model for blood donor.

**Fig 29:**
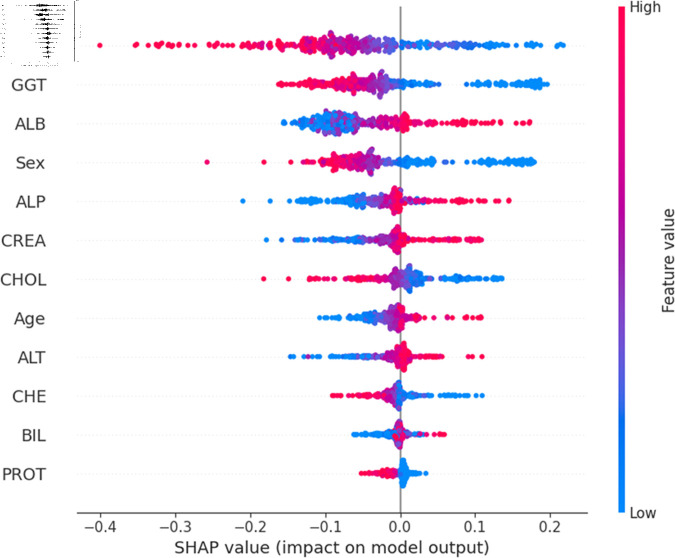
Explainability analysis of support vector machine model for suspect blood donor.

**Fig 30:**
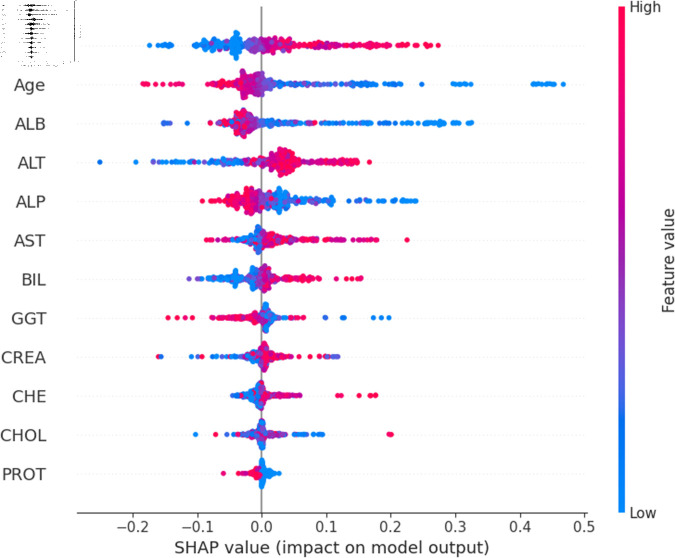
Explainability analysis of support vector machine model for hepatitis.

**Fig 31:**
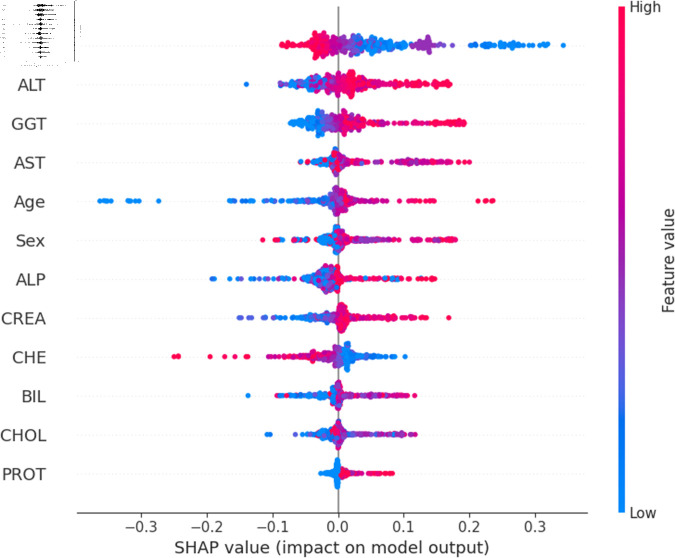
Explainability analysis of support vector machine model for fibrosis.

**Fig 32:**
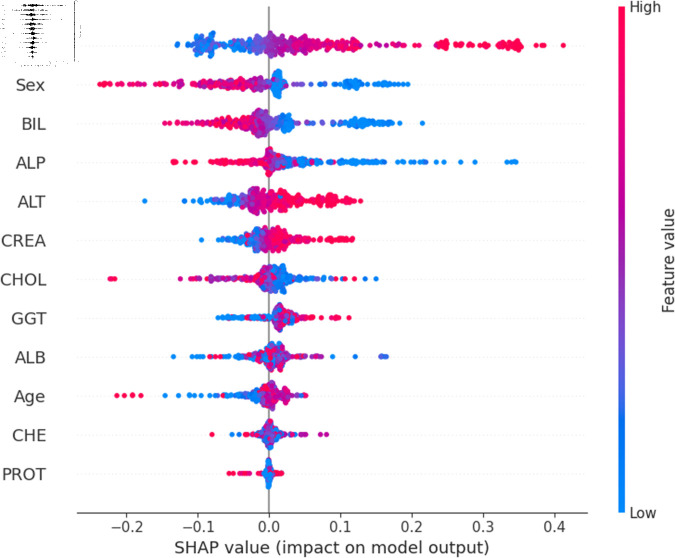
Explainability analysis of support vector machine model for cirrhosis.

The widely used surrogate method known as SHAP, or SHapley Additive exPlanations, explains the prediction of an observation by considering the contribution of each characteristic to that prediction. The SHAP analysis was carried out on the validation set of the dataset. To enhance interpretability, the study employed two visualization tools: variable importance with the summary plot (Figs 21 and 22 ) and summary plot of a specific target (Figs 23 to 32). As seen in Fig 22, Sex is the most influential one, and ALT, BIL, and AST are the first three features with the second most contribution to the prediction. In contrast, features like GGT, Age, ALB, and other remaining features show fewer contributions to the prediction. It can also be observed that the blue color (representing class 0) occupies most of the horizontal rectangle in the case of ALT which means has a greater impact on the classification of blood donors or class 0. In the case of our other highest-performing model (SVM), it can be seen (Fig 21) that the Aspartate aminotransferase (AST) feature contributes most to the model’s performance. ALT, ALB, and Sex have been the second most impactful features for Support Vector Machine performance. Features like ALP, GGT, CREA, and other remaining features have a little but substantial impact on model performance.

Although both models proved to be promising in terms of identifying patients with hepatitis or liver conditions, they have some distinctions in their prediction analysis which is demonstrated in Figs 21, and 22. The major differences can be noticed in the feature’s impact that has the greatest impact on the model’s output. Despite having these distinctions, ALT has a greater impact on the classification of blood donors or class 0 in both models.

For a more granular overview of the impact of each feature in classifying Hepatitis C and its variations, the summary plot of the two models has been demonstrated in Figs 23 to 32. Figs 23 to 27 show the features ranked according to their average absolute SHAP values, with each point on the Y-axis representing a feature. The X-axis represents the SHAP values, where negative values push the model prediction toward the lower stages of infection (the opposing class) and positive values make it toward the higher stages of infection (the impacted class). The color bar on the right side represents the value of features, where red indicates high values and blue indicates low values. According to Fig 25, patients with high AST levels (blue dots) are more likely to be infected with hepatitis. Overall, features like ALT, AST, GGT, ALB, and BIL play crucial roles in determining the severity of hepatitis infection. Similarly in Fig 30, it is depicted by the support vector machine that higher AST levels correlate with more severe stages of hepatitis followed by other liver function markers like ALT, ALB, and BIL.

Using SHAP, features importance in distinguishing the level of liver inflammation have been carried out effectively by the top-performing models. This applies whether the goal is to identify patients with a low risk of liver damage (Figs 23 and 28) or to mark infected patients with much more severe cases of hepatitis (Figs 27 and 32).

### 3.4 Discussion

Research in medical science requires the proper implementation of methodologies due to the sensitivity of this field. Thus the real world of machine learning models for medical data analysis, like the prediction of Hepatitis or classification between liver conditions can prove to be effective in this field. Here, the goal of our study was to assess appropriate machine learning models for hepatitis prediction. As previously stated, we have practiced three consecutive approaches to compute the effectiveness of multiple classification models. We preprocessed our data set to apply a clear data set to the models. The outcome of the initial evaluation indicates that Multilayer Perceptron outperformed every model in terms of predictive accuracy, achieving an accuracy of 99%. Besides, MLP, Random Forest, and SVM also showed competitive performance in other metrics. The findings suggest that MLP is well-suited for the classification task and may be used in the clinical area. Furthermore, the optimization of model performance provides a revised finding that suggests SVM be the top performer with high scores in almost every evaluation metric, from achieving an accuracy of 99.25% to the highest score in precision, recall, and f1-measure. We have utilized the hyperparameter tuning for optimization which brings change to the performance of every model used in the study. Moreover, the thoroughness of our model optimization process brings an additional novelty to the study as we implemented a well-validated technique by combining cross-validation with hyperparameter tuning. This pairing gives a more reliable estimate of model performance, dealing with the potential overfitting and ensuring the reliability of our findings. In contrast, Random Forest showed a similar performance except in Recall and F1-measure. Multilayer Perceptron provides almost constant performance due to the significant impact of chosen hyperparameters. Hyperparameter tuning brings a remarkable change in the performance of the AdaBoost Classifier which was especially profound as it gained an increased accuracy of 72.5% from 28% as well as a good score in other metrics. Contrary to the aforementioned approaches, Our ensemble approach exhibited a superior predictive performance achieving an accuracy of 99.51% and notable scores in precision which highlights its effectiveness in integrating diverse models for enhanced accuracy and reliability. Research in medical science requires the proper implementation of methodologies due to the sensitivity of this field. Thus the real world of machine learning models for medical data analysis, like the prediction of Hepatitis or classification between liver conditions can prove to be effective in this field. Here, the goal of our study was to assess appropriate machine learning models for hepatitis prediction. As previously stated, we have practiced three consecutive approaches to compute the effectiveness of multiple classification models. We preprocessed our data set to apply a clear data set to the models. The outcome of the initial evaluation indicates that Multilayer Perceptron outperformed every model in terms of predictive accuracy, achieving an accuracy of 99%. Besides, MLP, Random Forest, and SVM also showed competitive performance in other metrics. The findings suggest that MLP is well-suited for the classification task and may be used in the clinical area. Furthermore, the optimization of model performance provides a revised finding that suggests SVM be the top performer with high scores in almost every evaluation metric, from achieving an accuracy of 99.25% to the highest score in precision, recall, and f1-measure. We have utilized the hyperparameter tuning for optimization which brings change to the performance of every model used in the study. Moreover, the thoroughness of our model optimization process brings an additional novelty to the study as we implemented a well-validated technique by combining cross-validation with hyperparameter tuning. This pairing gives a more reliable estimate of model performance, dealing with the potential overfitting and ensuring the reliability of our findings. In contrast, Random Forest showed a similar performance except in Recall and F1-measure. Multilayer Perceptron provides almost constant performance due to the significant impact of chosen hyperparameters. Hyperparameter tuning brings a remarkable change in the performance of the AdaBoost Classifier which was especially profound as it gained an increased accuracy of 72.5% from 28% as well as a good score in other metrics. Contrary to the aforementioned approaches, Our ensemble approach exhibited a superior predictive performance achieving an accuracy of 99.51% and notable scores in precision which highlights its effectiveness in integrating diverse models for enhanced accuracy and reliability.

**Table 6:** Comparative analysis with literature.

Authors	Methods	Dataset Used	Acc. (%)	Prec. (%)	Sens. (%)	Spec. (%)	ROC-AUC (%)	F-measure (%)
Our study	SVM	Online HCV UCI dataset (5 classes, 12 features, 615 instances)	99.25	98.47	98.67	–	99.82	98.53
	RF		99	98.47	99	–	98.9	98.9
	Ensemble (RF, SVM, MLP)		99.51	99.54	99.5	–	99.82	99.51
Lilhore, Umesh Kumar, et al. [5]	HPM	Online HCV UCI dataset (4 classes, 29 features, 1756 instances)	96.82	98.93	99.13	–	–	97.54
Safdari, Reza, et al. [7]	RF	Online HCV UCI dataset (5 classes, 12 features, 615 instances)	97.29	96.28	90.99	82.6	99.8	93.42
Singh, Utkrisht, et al. [8]	LR	Dual dataset: Online HCV UCI dataset 1 (5 classes, 12 features, 615 instances), Online Hepatitis UCI dataset 2 (2 classes, 19 features, 155 instances)	94.3	55.81	57.14	–	–	59.63
Ma, Ling, et al. [10]	XGB	Online HCV UCI dataset (5 classes, 12 features, 615 instances)	91.56	90	98	–	–	98
Ahammed, Khair, et al. [11]	KNN	Online HCV UCI dataset (4 classes, 29 features, 1385 instances)	94.4	–	94.4	98.14	96.27	94.4
Syafaâ, Lailis, et al. [12]	NN	Online HCV UCI dataset (10 features, 73 instances)	95.12	88	82	–	–	–
Li, Tzuu-Hseng S., et al. [13]	RF, LR, ABC	Online UCI HCV dataset (4 classes, 14 features, 615 instances)	94.5	–	–	–	–	–

The comparative analysis of our study with other studies based on a similar data set reveals several key insights. As we can see in Table 6, the Random Forest model implemented by Safdari, Reza, et al. demonstrated strong performance with an accuracy of 97.29% and a ROC-AUC of 99.8% [7]. However, it exhibited slightly lower sensitivity and specificity compared to our SVM model. Lilhore, Umesh Kumar, et al. gained a lower accuracy of 96.82% with their Hybrid Prediction Model (HPM) approach [5]. Although their hybrid model performed well in terms of precision and sensitivity, it was outperformed by the SVM model of our study in terms of predictive accuracy. Another interesting study accomplished by Singh, Utkrisht, Mahendra Kumar Gourisaria, and Brojo Kishore Mishra [8] has shown the use of the Logistic Regression model which obtained a lower accuracy of 94.3% and a significantly lower precision of 55.81% suggesting that LR may not be effective as other complex models like SVM or random forest. As seen in Table 6, other studies including Ma, Ling, et al [10], Ahammed, Khair, et al [11] also showed promising results but slightly lower than the performance of the SVM model of our study.

A significant notable study conducted by Hira, Navdeep demonstrated impressive and complementary results with the SVM model for the prediction of hepatitis achieving an accuracy of 99.1% and a perfect sensitivity score of 100% [9]. Despite using the same grid search method (GridSearchCV) with 5 fold, a little dissimilarity bolsters our study than their study. The main reason behind this subtle difference could be figured out from the best parameters of their grid search approach: [‘C’: ‘10’, ‘gamma’: ‘0.0001’, ‘kernel’: ‘linear’]. Hence, using the Radial Bass Function or RBF as a kernel for the SVM algorithm proved more efficient than the ’linear’ kernel function. While our study achieved a slightly higher accuracy of 99.25%, both studies indicate the effectiveness of the SVM model for hepatitis prediction.

Our study has some limitations. First of all, the size of the data set may be sufficient for initial analysis but not good enough to conduct such a clinical prediction study. The more data we can train, the better the generalization of a machine-learning model’s performance. A data set’s restricted size might give rise to critical issues like overfitting or underfitting, model complexity, and so on. For this reason, future studies should focus on expanding the data collaboration with multiple healthcare institutions or leveraging existing large-scale healthcare databases to provide representative data sets. In addition to that, future research should explore more effective methodologies and approaches to construct better ML models for prediction analysis.

## 4 Conclusion

One of the main causes of death in the world today is liver disease. Hepatitis, Fibrosis, and Cirrhosis are considered to be the stages of chronic liver disease which then leads to the permanent failure of the liver. Early diagnosis often prevents permanent damage and timely classification boosts the process of diagnosis. Our study centered on the prediction of Hepatitis and the classifying of liver diseases. Based on the purpose of finding an accurate predictive model, Several machine-learning models specifically classifiers were applied to the HCV [14] data set and evaluated in terms of performance metrics e.g. accuracy, and precision. The data set underwent a thorough methodology process and the final observation regarding the performance of models stated that the optimal choice for precise prediction was the Support Vector Machine (SVM), which had an accuracy of 99.25%. Besides that, our study also employed an ensemble method to obtain superior performance for classification tasks using diversification of the three best-performed models. The ensemble method resulted in a more stable performance by achieving an accuracy of 99.51%. The study successfully comes up with an insight into the explainability of a model specifically the feature importance of the model’s outcome. Moving forward, future research could benefit from expanding the dataset eliminating the limitations identified in this study. The incorporation of advanced and emerging technologies with the potential of machine learning can enable more comprehensive analysis and accurate prediction in future studies. In light of these findings, it becomes evident that machine learning has the potential to revolutionize disease prediction, paving the way to early diagnosis and ultimately preventing critical issues such as liver failure.
